# SAPHO-like bone scintigraphy in diffuse idiopathic skeletal hyperostosis: a case report

**DOI:** 10.3389/fmed.2026.1967844

**Published:** 2026-10-07

**Authors:** Xiaolei Wang, Yuwei Wang, Guangying Zhang, Hongxu Liu, Sheng-Guang Li, Chen Li, Yuying Zhang, Di Jin

**Affiliations:** 1 Weifang Nursing Vocational College, Weifang, Shandong, China; 2 Department of Emergency Internal Medicine, Weifang People’s Hospital, Weifang, Shandong, China; 3 Department of Thoracic Surgery, Weifang People’s Hospital, Weifang, Shandong, China; 4 School of Traditional Chinese Medicine, Beijing University of Chinese Medicine, Beijing, China; 5 Department of Rheumatology and Immunology, Peking University International Hospital, Beijing, China; 6 Department of Dermatology, Tianjin Institute of Integrative Dermatology, Tianjin Academy of Traditional Chinese Medicine Affiliated Hospital, Tianjin, China; 7 Department of Rheumatology, Weifang People’s Hospital, Weifang, Shandong, China

**Keywords:** bone scintigraphy, differential diagnosis, diffuse idiopathic skeletal hyperostosis, SAPHO syndrome, SPECT, sternoclavicular joint, whole-spine CT

## Abstract

**Background:**

Bone scintigraphy is sensitive to increased osteoblastic activity but is not disease specific. Diffuse idiopathic skeletal hyperostosis (DISH) may therefore be mistaken for synovitis, acne, pustulosis, hyperostosis, and osteitis (SAPHO) syndrome when uptake involves the anterior chest wall and spine.

**Case presentation:**

A 50-year-old woman presented with a 1-year history of painless left sternoclavicular prominence. She reported that her daughter had SAPHO syndrome with anterior chest wall pain and palmoplantar pustulosis. Whole-body 99mTc-MDP scintigraphy and single-photon emission computed tomography showed uptake in the left sternoclavicular joint and multiple cervical and thoracic vertebrae. However, the patient had no inflammatory pain, skin lesions, or marked systemic inflammatory response. A low-dose whole-spine CT demonstrated flowing anterolateral ossification across multiple contiguous thoracolumbar vertebrae with preserved disc spaces; a sacroiliac CT showed no erosive sacroiliitis. A sternoclavicular MRI showed no bone marrow edema. These findings supported DISH. During 3 years of follow-up, no skin disease or osteitis-related symptoms developed.

**Conclusion:**

DISH should be considered when SAPHO-like scintigraphy is discordant with the clinical findings and CT/MRI evidence. A six-step approach integrating symptoms, skin findings, metabolic and medication history, MRI, CT, and follow-up may help assess this discordance, but this has not been validated.

## Introduction

SAPHO syndrome is a rare clinicoradiologic entity characterized by variable combinations of synovitis, acne, pustulosis, hyperostosis, and osteitis (1, 2). The anterior chest wall, particularly the sternocostoclavicular region, is a classic site of involvement. Cutaneous and skeletal manifestations may be asynchronous, and some adult patients present without active skin lesions, which can complicate diagnosis (3). For this reason, suspected SAPHO syndrome is best approached as an integrated clinical and imaging diagnosis that also requires exclusion of mimics, including infection, malignancy, spondyloarthritis, retinoid-related hyperostosis, and diffuse idiopathic skeletal hyperostosis (DISH) (2, 3).

DISH is a systemic, non-inflammatory bone-forming disorder characterized by flowing ossification along the anterolateral spine and peripheral entheseal ossification (4–6). Resnick-type radiographic criteria include ossification across at least four contiguous vertebral levels, relative preservation of intervertebral disc height, and absence of sacroiliac erosion or fusion (4, 5). DISH is often associated with metabolic factors such as obesity, diabetes, dyslipidemia, hyperuricemia, and metabolic syndrome, although their absence does not exclude the diagnosis when the characteristic imaging pattern is present (7, 8).

99mTc-MDP bone scintigraphy reflects osteoblastic activity and bone turnover rather than SAPHO-specific inflammation. The classic SAPHO “bull's head” sign comprises uptake in the manubrium and both sternoclavicular regions (3, 9). Here, “SAPHO-like” describes uptake involving the sternoclavicular region and multiple spinal sites: it was unilateral at the left sternoclavicular joint, with right-sided thoracic uptake and no complete bull's head sign. The term describes an overlapping distribution, rather than a diagnostic criterion. Previous reports have described SAPHO mimicking malignancy and SAPHO with DISH-like radiologic features (3, 10). The present case adds the complementary diagnostic scenario of DISH accompanied by SAPHO-like scintigraphy in a patient whose reported first-degree family history made SAPHO a plausible initial consideration. It illustrates the value of correlating scintigraphy with clinical findings, computed tomography (CT), and magnetic resonance imaging (MRI).

## Case presentation

### Patient information and clinical assessment

A 50-year-old woman attended the rheumatology clinic because of a progressive, painless bulging prominence over the left sternoclavicular joint for 1 year. There was no local erythema, warmth, or tenderness. She denied having inflammatory back pain, chest wall pain, peripheral arthritis, fever, weight loss, acne, palmoplantar pustulosis (PPP), psoriasis, inflammatory bowel disease, or uveitis. The patient reported that her daughter had been diagnosed with SAPHO syndrome at another institution, manifesting as anterior chest wall pain with PPP. Her daughter was not under our care. This family history was the main reason the patient requested bone scintigraphy.

The patient had a normal body mass index (BMI) of 21.05 kg/m^2^ and no history of diabetes mellitus, hypertension, dyslipidemia, hyperuricemia, or gout. HbA1c was not measured. Serum urate was measured and was normal, but the numerical value was not recorded in the case summary. She had never smoked or consumed alcohol and reported no previous exposure to retinoids or other medications associated with hyperostosis. Laboratory testing did not suggest active systemic inflammation or infection: the erythrocyte sedimentation rate was 15 mm/h, C-reactive protein was 5.3 mg/L, white blood cell count was 4.6 × 10^9^/L, hemoglobin was 144 g/L, and platelet count was 178 × 10^9^/L. HLA-B27 testing was not pursued because she had no back pain or other symptoms of spondyloarthritis, and subsequent imaging did not support that diagnosis. A dermatologic examination revealed no acne, PPP, or psoriasis.

A physical examination confirmed enlargement of the left sternoclavicular region but showed no inflammatory arthritis, enthesitis, dactylitis, spinal motion limitation, or active dermatologic lesions. Taken together, the clinical and laboratory profile did not support clinically active SAPHO syndrome or inflammatory spondyloarthritis, although the family history warranted careful imaging correlation.

### Imaging and diagnosis

Whole-body 99mTc-MDP bone scintigraphy showed increased radiotracer uptake in the left sternoclavicular joint and on the right side of the T7, T8, and T10 vertebrae (Figures 1A,B). Single-photon emission computed tomography (SPECT) imaging demonstrated uptake in the left sternoclavicular joint and the C7 and T5–T9 vertebrae. In the context of the reported first-degree family history of SAPHO syndrome, this distribution raised concern for SAPHO-like osteoarticular involvement. However, the absence of inflammatory pain, skin lesions, and tenderness at the scintigraphic hot spots created a clear clinical-imaging discordance.

**Figure 1:**
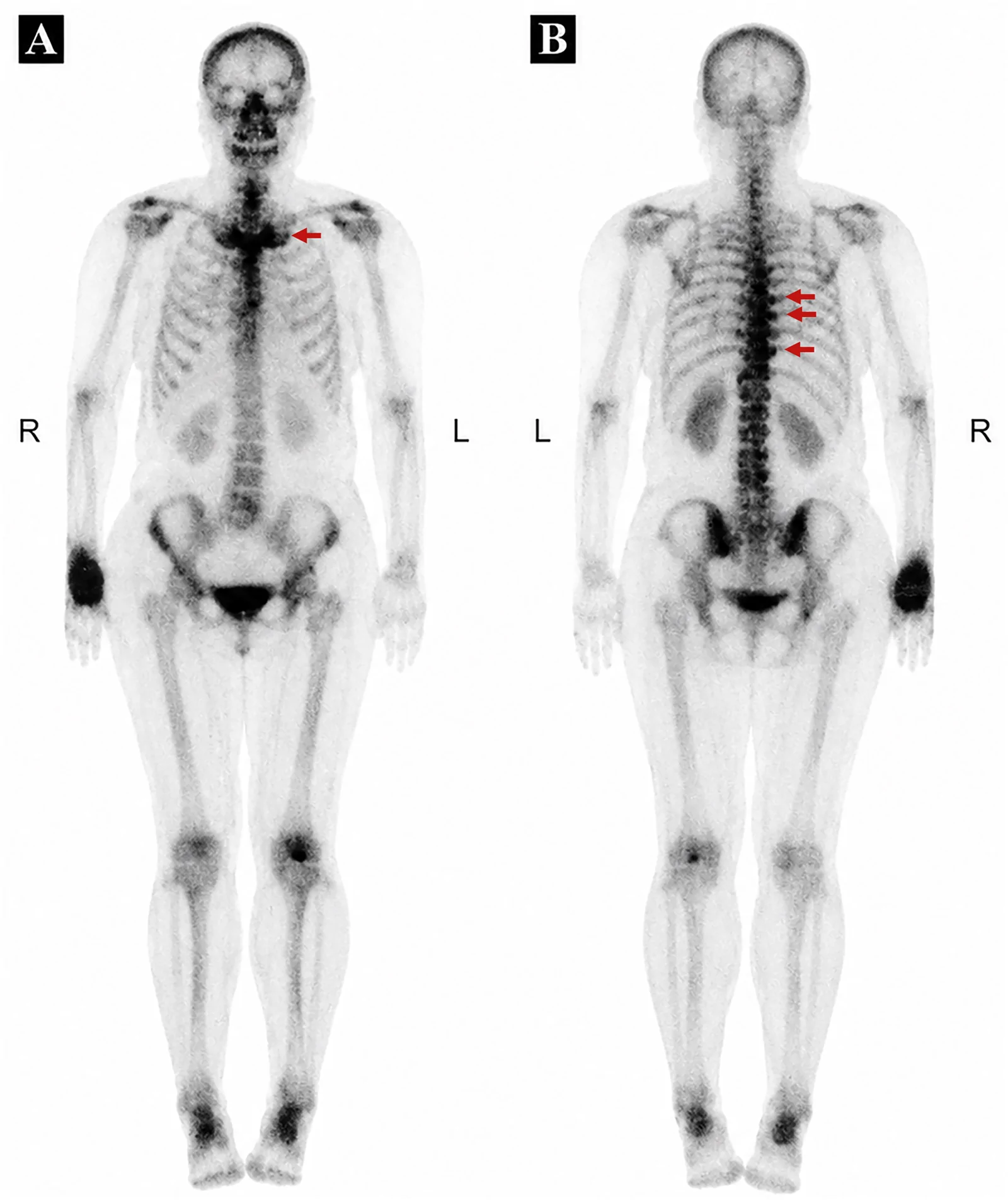
Whole-body bone scintigraphy showing SAPHO-like uptake. **(A)** The red arrow marks increased radiotracer uptake in the left sternoclavicular joint. **(B)** The red arrows mark increased uptake on the right sides of T7, T8, and T10.

A low-dose whole-spine CT was performed to define the anatomic basis of the tracer uptake. Coronal and sagittal reconstructions showed flowing anterolateral ossification along multiple contiguous thoracic and lumbar vertebrae, with preservation of intervertebral disc spaces and no destructive discovertebral lesions (Figures 2A,B). A CT of the sacroiliac joints showed no erosions, ankylosis, or destructive sacroiliitis (Figure 2C). An MRI of the sternoclavicular region demonstrated enlargement of the left sternoclavicular joint compared with the right side, without bone marrow edema or other features of active osteitis (Figure 2D). These findings fulfilled the diagnostic criteria for DISH and did not support SAPHO syndrome, ankylosing spondylitis, or another axial spondyloarthritis.

**Figure 2:**
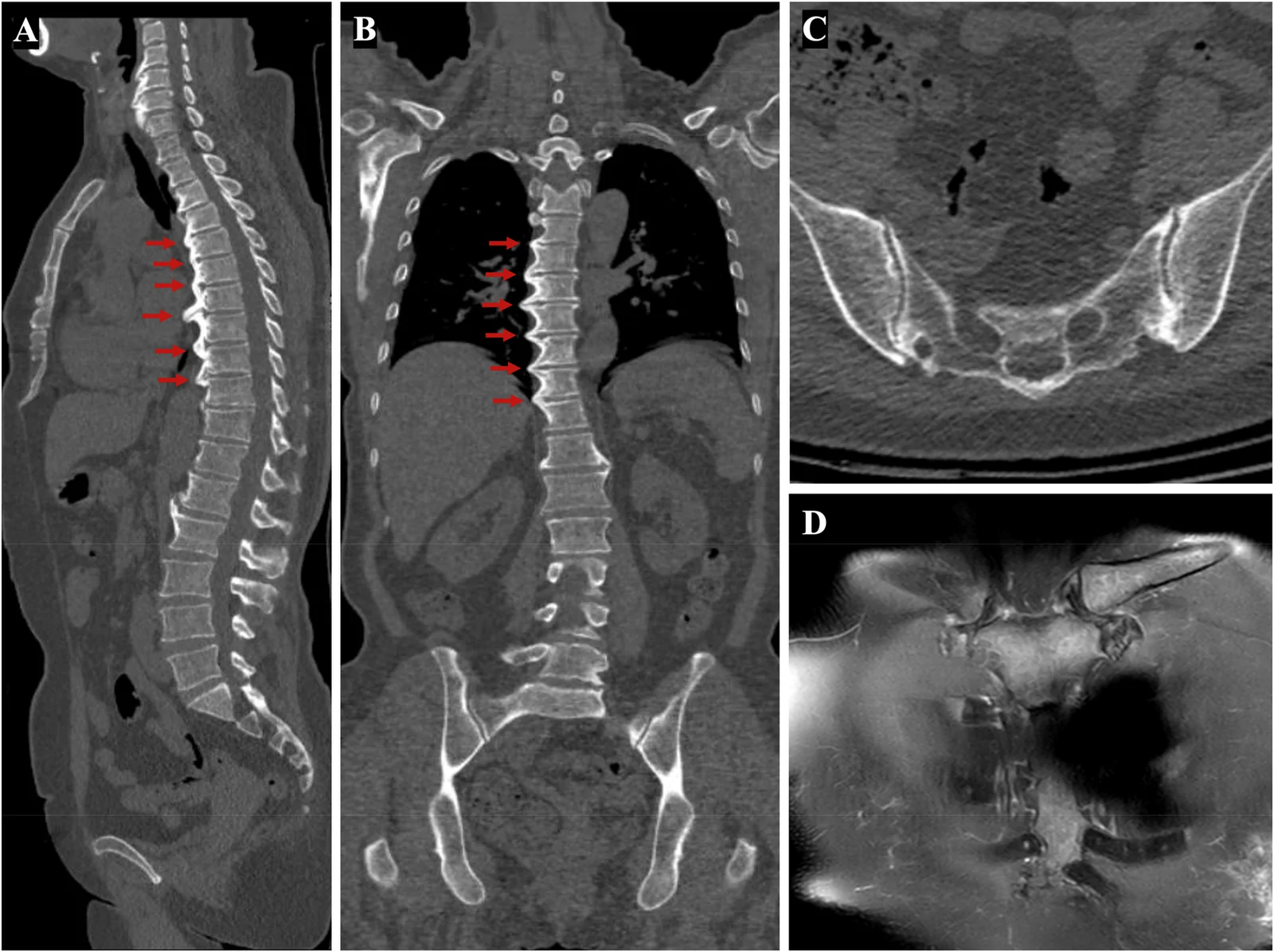
Low-dose CT and MRI findings supporting DISH. Sagittal **(A)** and coronal **(B)** CT images show flowing anterolateral ossification (red arrows) with preserved intervertebral disc spaces. **(C)** A sacroiliac CT shows no erosions, destruction, or ankylosis. **(D)** An MRI shows enlargement of the left sternoclavicular joint without bone marrow edema.

### Management and follow-up

Because the patient had no clinically significant pain or inflammatory manifestations, no NSAIDs, corticosteroids, disease-modifying antirheumatic drugs, biologics, or bisphosphonates were prescribed for SAPHO-like disease. She was advised to maintain mobility and was followed up clinically.

During 3 years of follow-up, the sternoclavicular prominence persisted without functional limitation, and she developed no bone pain, arthritis, acne, PPP, psoriasis, or other features of SAPHO syndrome. The key clinical, laboratory, and imaging findings relevant to the differential diagnosis between SAPHO syndrome and DISH are summarized in Table 1. The table shows how an initially plausible SAPHO concern was resolved by the concordant absence of inflammatory features and the presence of classic DISH morphology on CT.

**Table 1:** Diagnostic clues that prevented an overdiagnosis of SAPHO syndrome and supported DISH in the present case.

Feature	Present case	Diagnostic relevance
Age/sex	50-year-old woman	Although DISH is more common in men, it can occur in women after midlife
Main symptom	Painless left sternoclavicular prominence for 1 year	Absence of inflammatory anterior chest wall pain argued against clinically active SAPHO syndrome
Family history	Patient-reported diagnosis of SAPHO syndrome with palmoplantar pustulosis in her daughter	Raised pretest suspicion for SAPHO syndrome but was not diagnostic
BMI	21.05 kg/m^2^	No obesity-related metabolic risk factor for DISH
Diabetes mellitus/HbA1c	No history of diabetes mellitus; HbA1c not measured	Glycemic characterization was incomplete
Hypertension	No known history of hypertension	No reported hypertension; this does not establish a complete metabolic assessment
Dyslipidemia	No known history of dyslipidemia	An unremarkable history does not establish a normal lipid profile
Hyperuricemia/gout	No history of hyperuricemia or gout; serum urate measured and normal, numerical value not recorded in the case summary	No biochemical hyperuricemia was identified
Smoking/alcohol history	Never smoker; no alcohol consumption	General clinical characterization
Drug exposure	No retinoid exposure	Excludes retinoid-related hyperostosis as a SAPHO/DISH mimic
ESR	15 mm/h	No evidence of active systemic inflammation
CRP	5.3 mg/L	No marked systemic inflammatory response
CBC	WBC 4.6 × 10^9^/L; hemoglobin 144 g/L; platelets 178 × 10^9^/L	No evidence of infection, anemia, thrombocytosis, or systemic inflammatory response
HLA-B27	Not tested because symptoms and subsequent imaging did not suggest spondyloarthritis	HLA-B27 status remains unknown
Dermatologic findings	No acne, palmoplantar pustulosis, or psoriasis	Argued against clinically active SAPHO syndrome or psoriasis-associated spondyloarthritis
Whole-body bone scintigraphy	Increased uptake in the left sternoclavicular joint and right side of T7, T8, and T10	SAPHO-like distribution; potential source of diagnostic confusion
SPECT findings	Increased uptake in the left sternoclavicular joint and C7, T5-T9 vertebrae	Reinforced initial concern for SAPHO-like osteoarticular involvement
“Bull's head” sign	Absent	The classic pattern was absent; this does not exclude SAPHO syndrome
Whole-spine CT	Flowing anterolateral ossification across multiple contiguous thoracic and lumbar vertebrae with preserved disc spaces	Decisive structural feature supporting DISH
Anterior longitudinal ligament ossification	Present	Strongly supported DISH
Sacroiliac joint CT	No erosion, destruction, or ankylosis	Argued against ankylosing spondylitis or other axial spondyloarthritis
MRI findings	Left sternoclavicular enlargement without bone marrow edema or active osteitis	Absence of active osteitis argued against SAPHO syndrome
Resnick criteria for DISH	Fulfilled	Confirmed the diagnosis of DISH
Three-year follow-up	No bone pain, arthritis, acne, palmoplantar pustulosis, or psoriasis	Supported a non-inflammatory DISH phenotype rather than evolving SAPHO syndrome

Although the patient had SAPHO-like scintigraphic abnormalities and a patient-reported first-degree family history of SAPHO syndrome, the absence of inflammatory pain, dermatologic manifestations, systemic inflammation, bone marrow edema, and sacroiliac joint damage, together with characteristic flowing spinal ossification on CT, supported a final diagnosis of DISH.

BMI, body mass index; CBC, complete blood count; CRP, C-reactive protein; CT, computed tomography; DISH, diffuse idiopathic skeletal hyperostosis; ESR, erythrocyte sedimentation rate; HbA1c, glycated hemoglobin; MRI, magnetic resonance imaging; PPP, palmoplantar pustulosis; SAPHO, synovitis, acne, pustulosis, hyperostosis, and osteitis; SPECT, single-photon emission computed tomography; WBC, white blood cell count.

The patient reported that her main concern was having the same disease as her daughter. After CT and MRI clarified the diagnosis as DISH without active inflammatory osteitis, she felt reassured and preferred observation rather than medication. She remained satisfied with conservative management throughout follow-up.

## Discussion

This case illustrates a practical diagnostic pitfall in rheumatology: a SAPHO-like distribution on bone scintigraphy may appear persuasive when the sternoclavicular region and spine are involved, but it must be reconciled with the patient's clinical phenotype and cross-sectional imaging. As summarized in Table 1, the reported first-degree family history and scintigraphic abnormalities made SAPHO a reasonable initial consideration. The final diagnosis shifted toward DISH because the patient had no inflammatory pain, no acne or PPP, no meaningful systemic inflammatory response, no MRI bone marrow edema, no sacroiliac erosion, and characteristic flowing anterolateral ossification on CT.

The biological basis of tracer uptake explains the overlap. 99mTc-MDP accumulates at sites of osteoblastic activity and bone remodeling; it does not distinguish sterile inflammatory osteitis from non-inflammatory enthesopathic ossification. Tracer uptake at an ossification site does not by itself establish an inflammatory cause. In SAPHO syndrome, by contrast, uptake usually reflects a combination of osteitis and hyperostosis and is often accompanied by pain, swelling, sclerosis, erosion, or MRI marrow edema (3, 9, 11). CT and MRI were therefore decisive in the present case: CT established the structural pattern of DISH, whereas MRI showed no active osteitis at the clinically relevant sternoclavicular site. A DISH case evaluated with SPECT/CT showed mild right-sided lumbar uptake (12). This observation does not establish a characteristic scintigraphic pattern at extraspinal entheses.

The sternoclavicular presentation is also instructive. Anterior chest wall involvement is central to SAPHO syndrome, but DISH is not confined to the spine and may involve extraspinal, entheseal, and ligamentous sites (6). Mechanical or entheseal bone formation is a possible explanation for the painless sternoclavicular prominence and uptake in this patient, but the tissue responsible for the uptake was not established. The unilateral painless swelling and lack of marrow edema made active SAPHO less likely. Absence of the bull's head sign alone does not exclude SAPHO syndrome (3).

Family history should be interpreted as a modifier of pretest probability and not as a diagnostic substitute. Familial aggregation of SAPHO syndrome, including involvement of first-degree relatives, has been reported, supporting a possible genetic or shared environmental contribution in some patients (13). In the present case, the patient-reported diagnosis of SAPHO in her daughter appropriately increased clinical vigilance. Nevertheless, there is no established evidence that SAPHO in a first-degree relative predisposes to maternal DISH. The reported occurrence of SAPHO in the daughter and DISH in the mother may be coincidental; this case cannot establish a biological link.

Although DISH is frequently associated with obesity, diabetes, dyslipidemia, hyperuricemia, and metabolic syndrome (7, 8), this patient had a normal BMI and no known history of these metabolic conditions. Serum urate was normal, but HbA1c was not measured, and therefore, glycemic characterization was incomplete. Metabolic factors are supportive but not required for diagnosis when characteristic flowing spinal ossification is present.

The management implications are clinically relevant. Mislabeling asymptomatic DISH as SAPHO could lead to unnecessary anti-inflammatory or immunomodulatory treatment, repeated imaging, and persistent disease-related anxiety. Conversely, recognizing DISH allowed reassurance, mobility advice, and conservative observation. The six-step approach in Box 1 may help assess SAPHO-like scintigraphy in a clinical context. It draws on the imaging literature and this single case and has not been validated as a diagnostic algorithm. Its diagnostic performance is unknown, and the steps should be adapted to the clinical findings rather than applied as a fixed testing sequence.

Box 1. A practical approach to SAPHO-like bone scintigraphy

1. Assess inflammatory pain and tenderness at the tracer-uptake sites.
2. Examine for current or previous palmoplantar pustulosis, acne, psoriasis, or hidradenitis suppurativa and ask about inflammatory bowel disease.
3. Review metabolic and medication history, including retinoid exposure; distinguish unavailable laboratory data from normal results.
4. Assess targeted MRI for bone marrow edema and other features of osteitis when inflammatory disease remains a concern.
5. Use CT to distinguish flowing ligamentous ossification from erosive or destructive lesions and assess disc spaces and sacroiliac structural changes.
6. Follow up the patient clinically and reassess if inflammatory pain, arthritis, or skin manifestations develop.

*This approach is unvalidated and does not replace clinical judgment.*

This report describes a single patient. Bone biopsy and genetic testing were not performed because the CT/MRI findings, absence of systemic features, and 3-year follow-up did not suggest malignancy, infection, or active inflammatory osteitis. HLA-B27 was not tested because the symptoms and subsequent imaging did not suggest spondyloarthritis; its status remains unknown. HbA1c was not measured, limiting glycemic characterization. Finally, the absence of SAPHO manifestations during 3 years of follow-up does not exclude their later development.

## Conclusion

In this patient, DISH was accompanied by SAPHO-like scintigraphic uptake in the sternoclavicular region and spine. In patients with SAPHO-like bone scintigraphy but no inflammatory pain, no dermatologic manifestations, and no MRI evidence of osteitis, DISH should be actively considered even when a reported family history initially increases suspicion for SAPHO syndrome. Low-dose whole-spine CT and targeted MRI provide essential specificity and may prevent an overdiagnosis of SAPHO syndrome and unnecessary treatment.

## Data Availability

The original contributions presented in the study are included in the article/Supplementary Material; further inquiries can be directed to the corresponding author/s.
